# Experiences and satisfaction of video follow up of children with paediatric gastrointestinal conditions linking tertiary centre with guardians and clinicians at the local hospital: a cross-sectional study

**DOI:** 10.1186/s12887-023-04475-3

**Published:** 2024-01-03

**Authors:** Ann-Marie Kassa, Niklas Nyström, Kajsa Waldenvik, Helene Engstrand Lilja

**Affiliations:** 1 Department of Women’s and Children’s Health, Uppsala University, Uppsala, Sweden; 2 Paediatric Surgery unit, Uppsala University Children’s Hospital, Uppsala, SE-751 85 Sweden; 3 Paediatric gastroenterology unit, Uppsala University Children’s Hospital, Uppsala, SE-751 85 Sweden

**Keywords:** Children, Follow up, Paediatric gastrointestinal conditions, Telemedicine, Videoconferencing

## Abstract

**Background:**

Children with complicated gastrointestinal conditions are dependent on follow up by tertiary care specialists throughout childhood to prevent and treat complications. In Sweden, paediatric surgical- and intestinal rehabilitation centres are centralised which means that many patients and guardians have to travel long distances to access tertiary referral centres. Our tertiary referral centre has developed a model of shared care with video conferences for follow up with our centre and the patient and guardians attending together with the responsible professionals at the local hospital. This study aimed to investigate the experiences and satisfaction with video follow-up visits (VFV) between a tertiary referral centre and guardians and clinicians at their local hospital.

**Methods:**

Eligible participants were families with children with oesophageal atresia, intestinal failure and intestinal motility disorders and their local clinicians attending VFV with our tertiary referral centre from 2015 to 2020. Questionnaires included fixed-response alternatives, a 6-point Likert scale and open questions.

**Results:**

Fifty-seven out of 102 families (56%) and 19 out of 27 local clinicians (70%) responded the questionnaires. In 68% of the VFV, two guardians attended compared to 35% in the physical visits. Of the guardians attending VFV, 82% lost ≤ half a working day and 91% attending physical visits lost ≥ one full working day. Median distance to the tertiary referral centre was 267 km and attending VFV avoided emissions of 7.2 metric tonnes of CO_2_. Of the guardians, 90% and of the clinicians 95% were satisfied with VFV. Advantages were avoidance of travelling and the participants shared the same information.

**Conclusions:**

VFV is an appropriate alternative to physical visits with a high grade of satisfaction among the guardians and clinicians. VFV was time-saving for the families and reduced CO_2_ emissions.

## Background

A significant proportion of children with complicated gastrointestinal conditions are dependent on follow up by tertiary care specialists throughout childhood to prevent and treat complications that could impair function and health-related quality of life. In Sweden, paediatric surgical- and intestinal rehabilitation centres are centralised which means that many patients and guardians have to travel long distances to ensure access to specialised health care provided by tertiary referral centres.

Telemedicine seems to be a suitable option to physical visits for children living far away from a tertiary referral centre. The use of telemedicine by means of video or telephone for communication between the treating hospital and the discharged patient/guardian has been described for follow up after complex neonatal surgery in infants and various paediatric surgical procedures [1–4]. Furthermore, it has been used for follow-up visits by children with medical complexity, children within various paediatric subspecialties and children with Home Parenteral Nutrition (HPN) [5–7].

Since 2015, our tertiary referral centre has developed a model of shared care with video conferences for follow up with our centre and the patient and guardians attending together with the responsible professionals at the local hospital. The frequency of video follow-up visits (VFV) in general depends on the condition of the patient. A stable child with intestinal failure is usually followed through VFV 2–3 times per year and once per year as a physical visit at the tertiary clinic. The regular follow up for oesophageal atresia (EA) through VFV could be at 3 and 6 months, 3–4 years, 7–8 years and 12 years and physical visit at the tertiary clinic at the age of one and 15 years. VFV is planned in between these time-points when needed.

To ensure the confidentiality of the patients’ personal information a particular video conference system is used with a virtual meeting-room linking two hospitals through a secure encrypted connection via hospital data servers.

Previous studies of follow up by telemedicine in the paediatric population are scarce and include a combination of follow up by telephone and video consultations. All of them have reported a high degree of family satisfaction and the saving of costs and time [3, 4, 6, 8–10]. The video conferences in all previous studies have been performed by the tertiary hospital and the patient/guardians in the patients’ home or at the local hospital. To our knowledge no previous studies have reported the results using our concept of a video conference with the tertiary referral centre and the local professionals attending the video conference together with the patients and guardians at the local hospital.

The aims of this study were to investigate the experiences and satisfaction with video follow-up visits between a tertiary referral centre and guardians and clinicians at their local hospital. Moreover, to investigate potential time optimisation for the guardians and CO_2_ emission reduction compared to physical visits at the tertiary referral centre.

## Methods

Those eligible for the study were families with children with oesophageal atresia, intestinal failure and intestinal motility disorders and their local clinicians participating in VFV with a multi-professional team at the University Children’s Hospital, Uppsala Sweden from 2015 to 2020. Our tertiary referral hospital serves a population of approximately two million inhabitants.

A questionnaire consisting of 48 items was constructed by the authors concerning the guardian’s experiences and satisfaction with their latest VFV. Thirteen items included fixed response alternatives regarding practical procedures at VFV, participation in VFV and physical follow-up visits, lost working hours, preferred type of follow up and the number of respondents. A six-point Likert-scale with 6 indicating strong agreement and 1 indicating strong disagreement was applied in 21 items concerning the guardians’ assessment of the child’s health condition and experiences of and satisfaction with the VFV. The questionnaire also included 14 items with open questions concerning the child’s characteristics, experiences of interaction in VFV, participants in VFV and physical visits, general aspects of VFV and physical follow-up visits with additional open lines for comments. The questionnaire was modified after having been tested on four guardians.

Similarly, a questionnaire aimed at the local clinicians consisting of 16 items concerning experiences and satisfaction with VFV with our tertiary referral centre was constructed. Fixed response alternatives were used in nine items concerning effects of VFV on their role as clinicians and on the cooperation with the tertiary referral centre, COVID-19 effect on VFV, viewpoints on continued VFV and preference for type of visits and participants. A six-point Likert scale ranging from 6 representing “very good” to 1 representing “very bad” was applied in three items concerning overall perception of the VFV and functioning of planning and technology. Four items consisted of open questions concerning experiences with respect to number of patients in VFV, viewpoints from the local professionals, suggestions for improvements, and additional open lines for comments.

Study information, a consent form and a questionnaire were sent out to eligible families and the responsible clinicians at the local hospitals during 2020. Families not fluent in written and spoken Swedish were excluded. By means of a telephone call, the guardians were asked regarding interest to participate and if they preferred to answer the questionnaire orally. Non-respondents received a maximum of two reminders.

Diagnoses and date of the latest VFV were obtained from the medical records. The shortest suggested route between the local hospital and our tertiary referral centre was obtained from Google maps [11] and used as an approximation of the saved travel distance on account of the video follow up. Emission of CO_2_ was calculated according to the website “Utsläppsrätt” [12].

The methodology and the questionnaires of the study were approved by the Swedish Ethical Review Authority, registration number 2020–02637. Informed consent was obtained from all parents or legal guardians, from the patients/participants aged 15 years or more and from the local clinicians participating in the study.

### Data analysis

Data was analysed using IBM SPSS Statistics for Windows (version 27, IBM Corp, Armonk, NY, USA). Numerical variables were summarised by median (range) and categorical variables were summarised by frequency (%). The free text from the open questions and comment fields was sorted into themes according to the content. The second highest scores (score 5) of the Likert scales were interpreted as “agree”/ “to a high degree”/ “satisfied”/“good” respectively.

## Results

Out of 102 invited families, 57 (56%) agreed to participate in the study. We received 54 questionnaires by mail and three were filled in by the researcher during a telephone call. The 57 responses represent 59 children since two families had two siblings followed up by video. The questionnaires were answered by one guardian in 26 families (46%) and by two in 31 families (54%). The median age of the children participating in the study was six years (1.4–17). Eleven (19%) of the children had a diagnosis of oesophageal atresia, 31 (52%) had intestinal failure and 17 (29%) intestinal motility disorders. The guardians’ participation in numbers of VFV was > 10 for seven families (12%), 6–10 for seven (12%), 3–5 for 15 (27%), 2 for eight (14%) and 1 for 20 families (35%). Thirty-three families (58%) had experiences of both VFV and physical visits at the tertiary referral centre. The median distance between the local hospital where the families participated in the VFV and the tertiary referral centre was 267 (77–847) km, distributed as < 200 km in 44%, 200–500 km in 35%, 501–800 in 7% and over 800 km in 12%.

The total distance was 18,844 km one way resulting in a 37,688 km round trip. Assuming that all families travelled by a mid-sized car using petrol or diesel and calculating a CO_2_ emission of 190.280 g CO_2_/km [12], the participants in our study have avoided emissions of 7.2 metric tonnes of CO_2_.

**Fig. 1:**
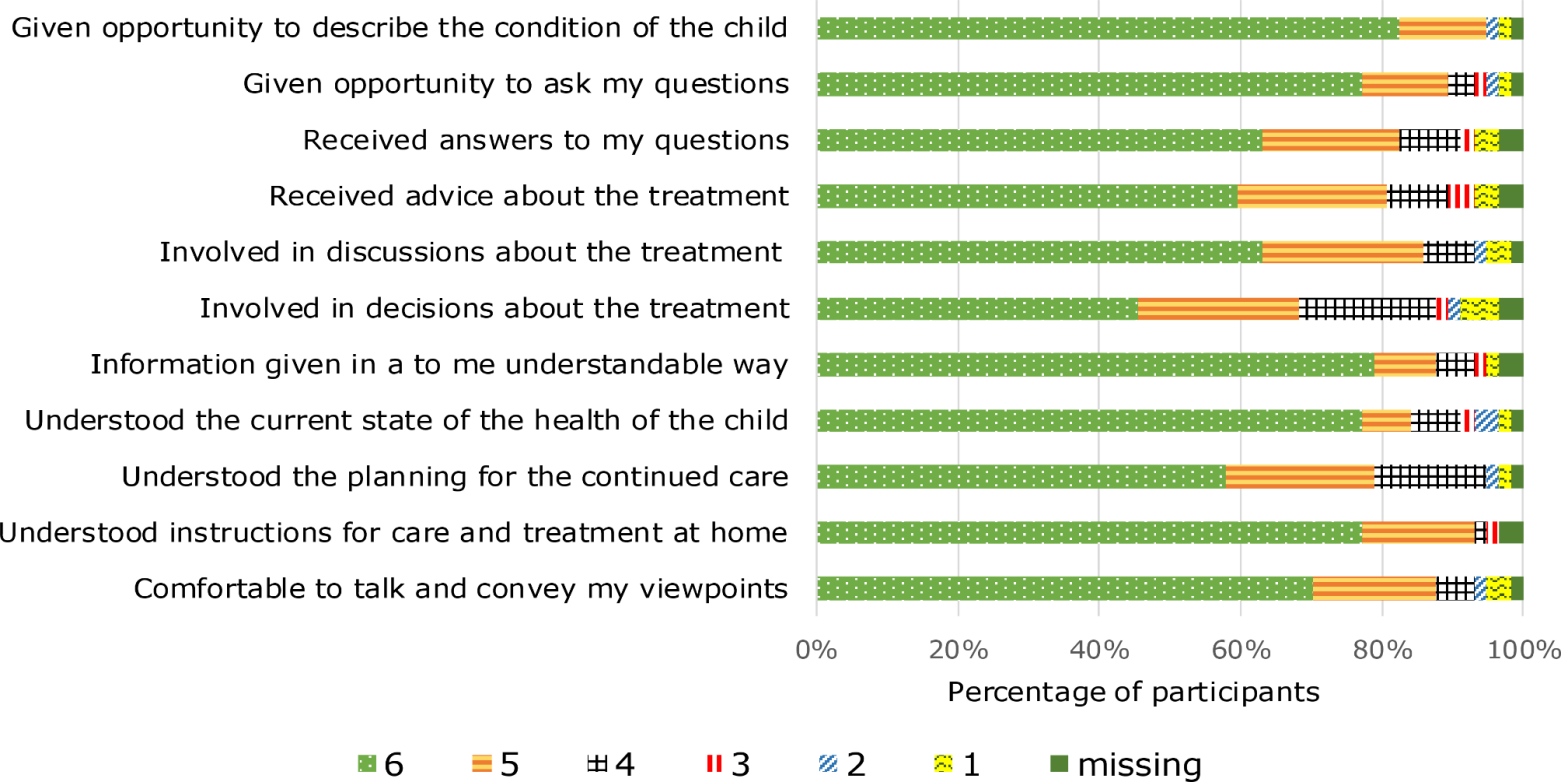
Guardians’ experience of interaction and communication during latest video follow-up visit (VFV). 6 = strong agreement, 1 = strong disagreement

Figure 1 illustrates the guardians’ experience of interaction and communication during their latest VFV. Between 79 and 95% of the guardians scored 5 or 6 on the statement items except for involvement in decisions about the treatment where 68% scored 5 or 6.

In rating of practical arrangements of VFV, 52 (91%) of the guardians scored 5 or 6 regarding that they could see all participants clearly and that they trusted the security of the internet connection, 50 (88%) that they could hear all participants and follow the discussion, 49 (86%) that the appointment invitation had been sent in good time and 44 (77%) that the VFV was of an appropriate length. On the question of whether they wanted to send information regarding the child’s condition and questions in advance, 17 guardians (30%) scored 5 or 6.

**Fig. 2:**
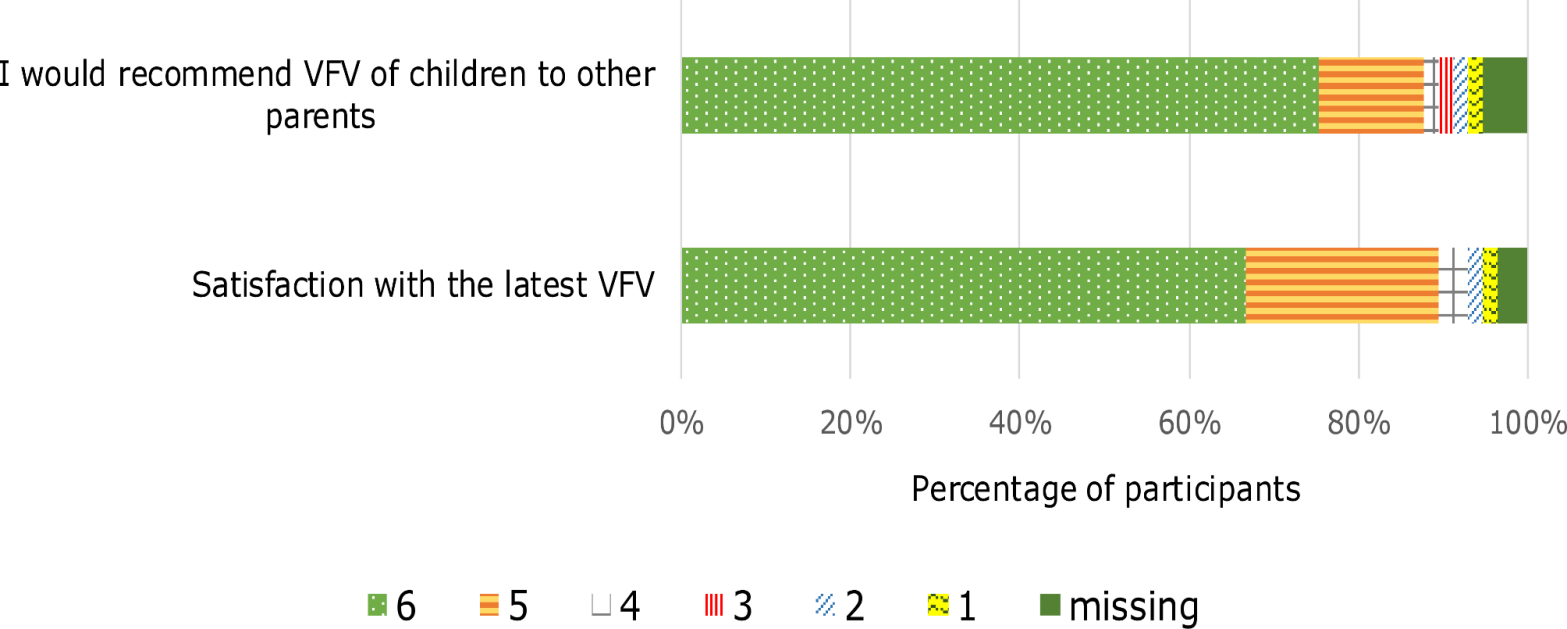
Guardians’ satisfaction with latest video follow-up visit (VFV) and recommendation to other parents. 6 = strong agreement, 1 = strong disagreement

Thirty-eight (67%) of the guardians were very satisfied (scored value 6) with their latest video follow up and 13 (23%) were satisfied (value 5). Fifty (88%) of the guardians strongly agreed or agreed (scored 6 or 5) that they would recommend video follow up to other parents. (Fig. 2).

In the open questions the guardians described their perception of the advantages and disadvantages of VFV with our tertiary referral centre as presented in Table 1. The advantages most often presented were the avoidance of long journeys and that all professionals and guardians involved shared the same information. The most frequently stated disadvantages of VFV was the lack of opportunity to perform a physical examination of the child and the occurrence of technical fuss.

**Table 1:** Guardians’ perceived advantages and disadvantages of video follow up

Advantages of video follow up	Disadvantages of video follow up
Avoidance of long journeys	Physical examination not possible
All involved receive the same information	Technical fuss
Avoidance of transporting a sick child	Impersonal
Close contact with specialists even at distance	Risk of overlooking signs of patient issues
Receiving answers to our questions	Shortage of time to get answers to questions
Easily accessible	Difficult to communicate with the child
Efficient	Easier to ignore parents
Both parents can participate	Sometimes communication between doctors with microphone muted
Timesaving	Lack of written documentation
Less absence from school and work	Limited opportunities to express their opinion
Avoidance of extra expenditure	
More frequent follow up possible	

From 39 (68%) of the families two guardians participated in VFV and from 20 (35%) families two guardians participated in physical visits to the tertiary referral centre. To participate in VFV, 79 (82%) of the total 96 guardians (1 + 2) had to be away from work half a day or less and nine (9%) needed to be away a whole day. To participate in a physical visit at the tertiary referral centre three (6%) of the total 53 guardians needed half a day or less, 48 (91%) one whole day or more whereof 23 (43%) more than one day. VFV was preferred by eighteen (55%) among the guardians who had experiences of both types of follow up, four (12%) preferred physical visits in the tertiary referral centre and seven (21%) preferred a combination of both. 12% did not answer the question.

Among the 27 letters sent out to the responsible clinicians in the local hospitals, 19 questionnaires were returned (70%). Thirteen of the clinicians had participated in VFV with less than 10 patients, four of 10 patients and two clinicians had participated concerning 20 patients.

**Fig. 3:**
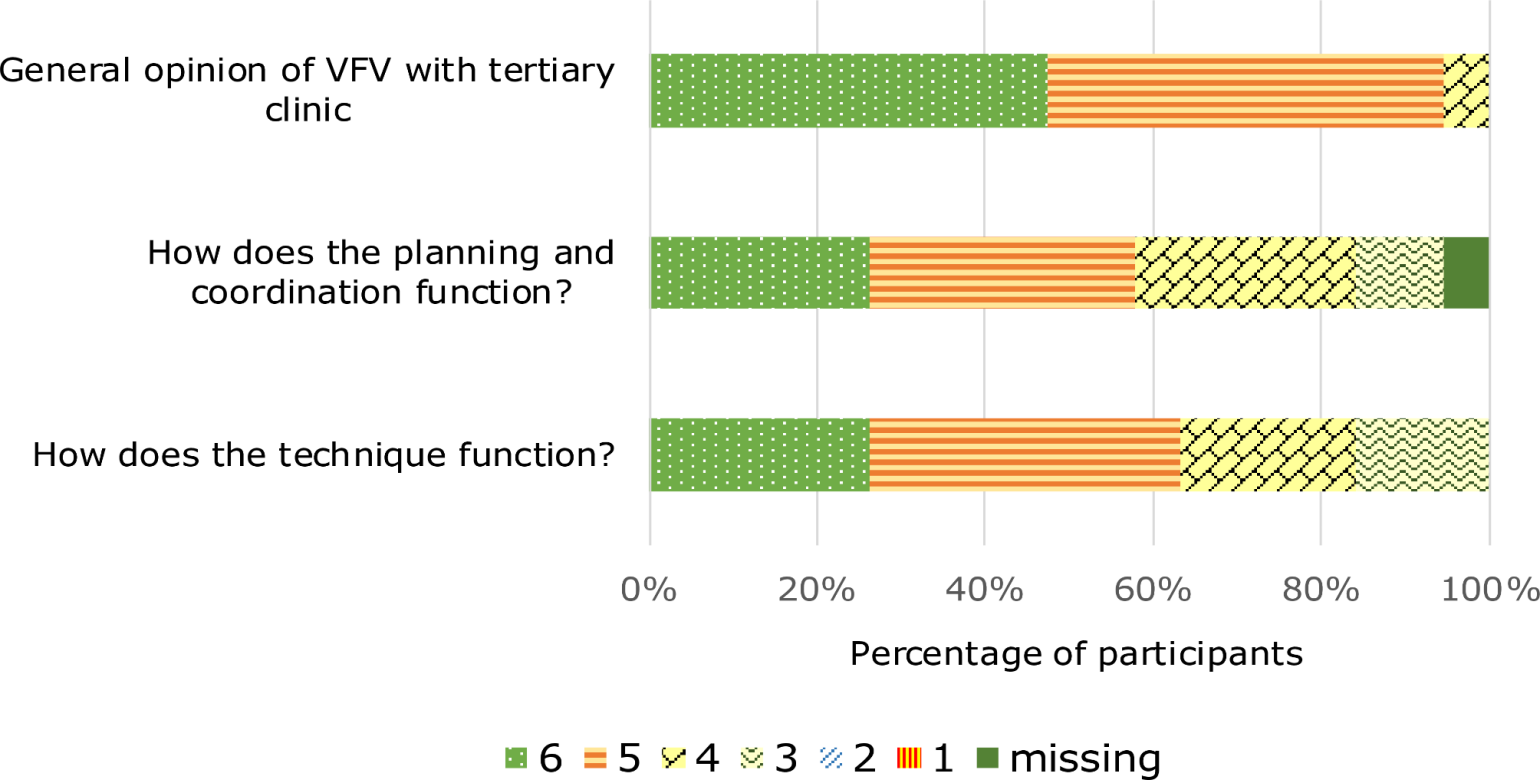
Local clinicians’ general opinion, experiences of planning, coordination and technology for video follow-up visits (VFV). 6-point Likert-scale; 6 = Very good, 1 = very bad

Figure 3 presents the clinicians’ rating of VFV at our centre. Nine (47%) of the clinicians scored 6 on the general opinion of VFV and nine (47%) scored 5. Eleven (58%) clinicians scored 5 and 6 on the planning and coordination and 12 (63%) scored 5 or 6 on the functioning of the technology.

Sixteen (84%) of the clinicians answered that VFV had improved the cooperation with the tertiary referral centre. Closer contact between the healthcare professionals in the two hospitals, greater consensus in assessments and planning, faster decisions and more efficient processing were other comments from the local clinicians. Also, patient-related motivation was commented on such as less travelling, more satisfied families and improved confidence in health care. Thirteen (68%) of the clinicians stated that the VFV had strengthened their role, being more involved and included in the treatment of the patients. Furthermore, the VFV had brought more evident shared responsibility, improved their competence, and facilitated treatment of more complicated patients. Moreover, follow up and planning improved since the clinician and family shared the same information.

All of the participating clinicians wanted to continue with VFV, twelve (63%) to increase them, none to decrease them and one clinician wanted to change them. VFV was chosen as the preferred type of follow up by 11 (58%) clinicians and five (26%) preferred both video and physical visits. Twelve (63%) of the clinicians preferred VFV with the professionals in our centre and with the patient and guardian present, one (5%) with only the guardian and four (21%) clinicians both with and without the patient and guardians.

Twelve (63%) clinicians did not think that the COVID-19 pandemic had influenced opportunities to use VFV. However, five (26%) of the clinicians had contrary opinions and provided comments such as “VFV has become more accepted”, “increased number of VFV which is positive” and “improved technology”. Two clinicians did not answer the question.

## Discussion

In this study we present the results using our concept of shared care with follow up by video with tertiary care specialists onsite and the local professionals attending together with the patients and guardians at the local hospital.

In general, all the guardians appreciated the VFV with approximately 90% satisfied with the latest VFV and would recommend this type of follow up to other guardians. The result is comparable to other studies reporting family satisfaction with follow up of paediatric patients by video consultations in 65–98% [3, 8, 9, 13, 14], and recommending postoperative video visits to other families [3]. Eighteen of the nineteen (95%) local clinicians in the present study rated their general opinion of VFV as good or very good which is a higher degree of satisfaction compared to clinicians in paediatric surgery who report overall satisfaction with video sessions at 73% and 74% [8, 14].

Telemedicine technologies include e-mail, telephone and video conferences. Video conferences seems favourable to follow up by telephone as they allows eye to eye contact between the participants and inspection of the patients. Telemedicine in the form of video conferencing is a necessary prerequisite to apply this model of shared care.

Most studies in telemedicine describe sessions between the clinician and the patients in their home [1, 3–7, 15] or in a remote tele-health or healthcare facility sometimes attended by a clinic nurse and rarely with the referring clinician present [2, 9, 13]. What makes our model favourable is that it also includes the professionals at the local hospital.

A specific benefit of VFV mentioned by both the guardians and the local clinicians was that all participants received information and took part in the discussion at the same time. The same medical information provided by the tertiary referral centre facilitates the management of the patients at the local hospital by avoiding misunderstandings that could jeopardise the patients’ and guardians’ confidence in the medical providers at the local hospital.

Another advantage of our model would be that the local responsible clinicians will be educated in the often rare medical and surgical conditions. Accordingly, the clinicians in our study described that the VFV increased their competence and provided support for them to handle more complicated patients. Similarly, Andrew et al. described how video follow up together with their general practitioner or local nurse present of adult patients after kidney transplantation could increase local knowledge and improve local management and care [16]. The advantage of VFV most often stated by the guardians was the avoidance of long journeys. Since paediatric tertiary care in Sweden is centralised, physical visits to these centres are time consuming and entail long journeys. In agreement with other studies the guardians attending VFV in the present study reported less time off work [4, 10, 13]. In addition to saving time for the families, environmental aspects should also be taken into account. The amount of CO_2_ emission avoided amounting to approximately seven metric tonnes corresponds to a domestic flight of approximately 400 km [17]. In comparison, in 2020, CO_2_ emissions were 4.18 metric tonnes per capita in Sweden [18].

The COVID-19 pandemic has resulted in the increased use of telemedicine in the world [6, 15, 19]. Even though the model applied in our centre was successfully implemented long before, the impact of the COVID-19 pandemic might have resulted in increased numbers of VFV and improved technology as suggested by the local clinicians.

The most often described disadvantage of VFV stated by the guardians in the present study was the inability for the tertiary care specialists to meet, examine and assess the child. Also, guardians studied by Lakshin et al. described disadvantages such as absence of physical examination of the child with the fear that some important medical information could be lost [15]. Similarly, other clinicians have reported the inability to perform physical examinations as a limitation to telemedicine [14, 16]. This would in our model still be a risk, but would be reduced on account of the local clinician’s presence in the VFV.

Furthermore, the participation of the child might be restricted in VFV since it may be difficult for the child to take part in the conversation and create contact with the healthcare providers as reported by Lakshin et al. [15]. Physical follow-up visits to the tertiary referral centre might be an important complement for the child to feel safe with the tertiary care specialists.

We believe that our model of VFV contributes to less risk of loss to follow up and improves the quality of care. It also increases the opportunity for both guardians to attend the follow up with their child. Still, it is important to consider that VFV may not completely replace physical visits.

A strength of our study is that it is the first study reporting a model of VFV with specialists at a tertiary referral centre and the patient and guardians attending with professionals at the local hospital.

A limitation of the study is the small sample size and inclusion of patients with a few diagnoses which might represent a selection bias. Furthermore, no information was obtained on the socioeconomic status of the guardians and economic loss from absence from work and travelling. The questionnaires were not validated but tested on four parents and reached a high response rate among the participant families.

## Conclusion

VFV is an appropriate alternative to physical visits with a high degree of satisfaction among the guardians and clinicians. VFV was time saving for the families, increased the possibility for both guardians to attend and reduced the CO_2_ emissions.

## Data Availability

Data privacy regulations prohibit deposition of individual level data to public repositories and the ethical approval does not cover public sharing of data for unknown purposes. Upon contact with the corresponding author (ann-marie.kassa@kbh.uu.se) an institutional data transfer agreement may be established and data shared if the aims of data use are covered by ethical approval.

## Abbreviations

HPN: Home Parenteral Nutrition
VFV: Video follow-up visits
